# Effect of *Rhus verniciflua* Extract on IgE-Antigen-Mediated Allergic Reaction in Rat Basophilic Leukemic RBL-2H3 Mast Cells and Passive Cutaneous Anaphylaxis in Mice

**DOI:** 10.1155/2019/6497691

**Published:** 2019-10-09

**Authors:** Hyun Ju Do, Yeo Jin Hwang, Hye Jin Yang, Kwang Il Park

**Affiliations:** ^1^Korean Medicine (KM)-Application Center, Korea Institute of Oriental Medicine (KIOM), 70 Cheomdan-ro, Dong-gu, Daegu 41062, Republic of Korea; ^2^Companion Diagnostics and Medical Technology Research, Daegu Gyeongbuk Institute of Science and Technology (DGIST), Daegu 711-873, Republic of Korea

## Abstract

*Rhus verniciflua* is widely known for its antioxidant, antibacterial, anticancer, and antiaging efficacy and *α*-glucosidase inhibition. This study was designed whether *Rhus verniciflua* extracts inhibit the IgE-antigen-mediated allergic reaction in RBL-2H3 mast cells, and it further investigated the Fc*ε*RI- and arachidonate-signaling by which *Rhus verniciflua* extracts exert its antiallergic effects. IgE-antigen-sensitized RBL-2H3 mast cells were investigated for the cytotoxicity of *Rhus verniciflua* extracts and *β*-hexosaminidase release, and inflammatory mediators (e.g., TNF-*α*, IL-4, IL-6, histamine, and PGD_2_) were then assessed. Additionally, we examined expressions of genes involved in arachidonate- and Fc*ε*RI-signaling pathway in RBL-2H3. *Rhus verniciflua* extracts inhibited *β*-hexosaminidase release and production of the inflammatory mediators in RBL-2H3. *Rhus verniciflua* extracts reduced amounts of histamine and expressions of Fc*ε*RI signaling-related genes such as Lyn and Syk and phosphorylation of extracellular signal-regulated kinase in mast cells. Finally, in late allergic responses, *Rhus verniciflua* extracts reduced PGD_2_ release and COX-2 and cPLA2 phosphorylation expressions from IgE-antigen-mediated mast cells. Lastly, 250–500 mg/kg RVE significantly attenuated the Ag/IgE-induced passive cutaneous anaphylaxis (PCA) reaction in mice. These findings provide novel information on the molecular mechanisms underlying the antiallergy properties of *Rhus verniciflua* extracts in Fc*ɛ*RI-mediated allergic reaction.

## 1. Introduction

*Rhus verniciflua* (RV) has been used in traditional Asian medicine in many countries including Korea and China to prevent gastritis, stomach cancer, atherosclerosis, aging, and hypertension and to promote blood circulation [1]. Recently, it has been shown to have antioxidant, antibacterial, and *α*-glucosidase-inhibition effects. Several studies reported that it inhibits proinflammatory cytokines and the vascular endothelial growth factor in fibroblast-like synoviocytes in in vivo models of rheumatoid arthritis [2]. It was also reported that RV inhibits the occurrence of atopic dermatitis-like lesions in animals with atopic dermatitis; RV has an anti-inflammatory effect on macrophages [3]. However, little has been known about the Fc*ε*RI-mediated cell signaling mechanism.

Mast cells contain Fc*ε*RI receptors, which are known as immunoglobulin E (IgE) receptors, in the plasma membrane [4]. When mast cells are activated, histamine, heparin, and proteases stored in the granules of these cells are released, producing chemical mediators such as leukotrienes and prostaglandins [5]. These chemical mediators expand the blood vessels within a short time, increasing their permeability. They also increase the vascular permeability and smooth muscle contraction, thereby inducing allergic reactions [5, 6]. Fc*ε*RIs on the mast cell surface consist of *α*, *β*, and *γ* subunits, of which the *β* and *γ* subunits are involved in cell signaling. When an allergen crosslinks with IgE, the receptor is phosphorylated by the Src-kinase combined with the *β* subunit, initiating cell signaling [7, 8]. Additionally, immunoreceptor tyrosine-based activation motifs (ITAMs) in the *β* and *γ* subunits are phosphorylated by Lyn kinase combined with the receptor [9]. The phosphorylated ITAMs provide binding pockets for Syk kinase, which plays a key role in cell activation, thereby activating Syk kinase. They then induce the activation of downstream signaling molecules [10]. In the initial phase of mast cell activation by an antigen, the most important signaling protein, Syk, is activated. This, in turn, activates various signaling molecules such as downstream signaling LAT and PKC [10, 11]. As a result, mast cells are activated, releasing histamines, prostaglandins, leukotrienes, and sensitivity factors. This activates the immune system, ultimately causing inflammation and allergic responses [12]. As explained, the study of the cell signaling mechanism is a highly important part of the study of allergic diseases.

We assumed that *Rhus verniciflua* extract (RVE) can demonstrate inhibitory effects on the allergic reaction of mast cells, which are mediated by Fc*ε*RIs. Therefore, we measured the *β*-hexosaminidase activity and assessed the degree of degranulation of Fc*ε*RI-mediated RBL-2H3 mast cells, in order to examine the antiallergic effect of RVE. Then, the effect of RVE on inflammatory cytokines such as TNF-*α*, IL-4, and IL-6 was assessed in RBL-2H3 mast cells. Finally, the effect of RVE on the antiallergic mechanism associated with the Fc*ε*RI signaling was investigated.

## 2. Materials and Methods

### 2.1. Chemicals and Reagents

HPLC-grade methanol was purchased from Fisher (Pittsburgh, PA, USA) and HPLC-grade acetic acid obtained from Sigma-Aldrich Co. (St. Louis, MO, USA). Ultrapure water was prepared by using the Puris-Evo UP water system with Evo-UP Dio VFT and Evo-ROP Dico20 (Mirae ST Co., Ltd., Anyang, Gyeonggi-do, Korea). Ultrapure water (UW) was prepared with a resistivity of 18.2 MΩ·cm^−1^ (Puris, Esse-UP water system, Mirae St Co., Anyang, Korea). Gallic acid was obtained from Tokyo Chemical Industry Co., Ltd. (Tokyo, Japan). Protocatechuic acid, methyl gallate, caffeic acid, ethyl gallate, and quercetin were purchased from Sigma-Aldrich (St. Louis, MO, USA). Butein was obtained from Faces Biochemical Co., Ltd. (Wuhan, China). The purity of all standards was above 97%. Minimum essential medium alpha modification (MEM-*α*), Dulbecco's phosphate-buffered saline (DPBS), fetal bovine serum (FBS), and antibiotics were purchased from GE Healthcare Life Sciences (Hyclone, UT, USA). Dinitrophenyl-human serum albumin (DNP-HSA), DNP-immunoglubulin E (DNP-IgE), 4-methyl-umbellyferyl-N-acetyl-*β*-d-glucosaminidase (p-NAG), dimethyl sulfoxide (DMSO), and dexamethasone were acquired from Sigma-Aldrich (MO, USA). Antibodies were obtained from Cell Signaling (MA, USA). The ethanolic extracts of *Rhus verniciflua* (RVE) were produced in Korea Institute of Oriental Medicine.

### 2.2. RVE Preparation

RV was obtained as a dried herb from Yeongcheon Oriental Herbal Market (Yeongcheon, South Korea) and was authenticated by the Korean Medicine Application Center, Korea Institute of Oriental Medicine. RV (50 g) was extracted using a 70% ethanol at 40°C for 24 h in a shaking incubator. Then, the extraction was filtered using a 150 mm filter paper and concentrated using a rotary vacuum evaporator (Buchi, Tokyo, Japan). The sample was freeze-dried and stored in desiccators at 4°C until use. The RVE powder was dissolved in 50% DMSO for the experiments.

### 2.3. HPLC Analysis of RVE

The seven standard stock solutions were prepared by dissolving accurately weighed in 100% methanol (1000 *μ*g/mL), respectively. The seven standards were mixed from stock solutions and then diluted at final concentrations of 100 *μ*g/mL, respectively. RV 70% ethanol extracts (20 mg/mL) were prepared in 100% methanol. Then, the standard solutions and sample were filtered through a 0.22 *μ*m membrane filter (Whatman International ltd., Maidstone, UK) prior to injection into the HPLC-DAD system. The injection volume was 5 *μ*L.

To identify seven components in RV, high-performance liquid chromatography (HPLC) analysis was performed using a Dionex UltiMate 3000 system (Dionex Corp., Sunnyvale, CA, USA) equipped with a binary pump, an autosampler, a column oven, and a diode array UV/VIS detector (DAD). System control and data analysis were processed with Dionex Chromeleon. The separation was carried out using a Xbridge C18 column (250 × 4.60 mm, 5 *μ*m, Waters, Milford, MA, USA), and the column oven temperature was kept at 40°C. The mobile phase consisted 2% acetic acid (v/v) in water (A) and methanol (B). The gradient elution system, to improve the chromatographic separation capacity, was programmed as follows: 5–20% B, 0–10 min; 20–35% B, 10–50 min; 35–50% B, 50–70 min; 50% B, 70–75 min; 50–80% B, 75–80 min; 80% B, 80–85 min; 80–5% B, 85–86 min; 5% B, 86–95 min at a flow rate of 1.0 mL/min. The detection wavelengths for seven components were set at 230, 254, 280, and 360 nm.

### 2.4. Cell Culture

RBL-2H3 mast cell line was acquired from the American Type Culture Collection (Manassas, VA, USA) and grown in MEM-*α* medium with 10% FBS and 1% antibiotics (100,000-Unit/L penicillamine and 100 mg/L streptomycin) in a humidified atmosphere of 95% air and 5% CO_2_ at 37°C. The growth medium was replenished every two days.

### 2.5. Cell Viability

Prior to the experiments, 2 × 10^4^ cells were seeded on a 96-well plate and grown to confluence overnight. The cells were rinsed with fresh DPBS and cultured in MEM-*α* with 0.1 *μ*g/mL DNP-IgE for 24 h. The cells were pretreated with various concentrations of RVE (100, 300, and 500 *μ*g/mL) and/or 100 nM dexamethasone for 1 h and treated with 0.1 *μ*g/mL DNP-HSA for 4 h. Cell viability was analyzed after the addition of 0.5 mg/mL MTT reagent in each well and an another additional incubation for 40 min at 37°C. After removal of the medium, cells were lysed with DMSO. The cell viability was measured using a microplate reader at 570 nm.

### 2.6. Measurement of *β*-Hexosaminidase

RBL-2H3 mast cells (2 × 10^4^/wells) were cultured into 96-well plate and preincubated with RVE. After 4 h of DNP-HAS (0.1 *μ*g/mL) treatment, cell culture supernatant of each well was allowed to react with 4-methyl-umbellyferyl-N-acetyl-*β*-d-glucosaminidase (10 mM p-NAG) for 1 h at 37°C. The reaction was terminated by adding sodium carbonate buffer (0.1 M, pH 10.0), and the absorbance was measured with a microplate reader at 405 nm.

### 2.7. Measurement of TNF-*α*, IL-6, IL-4, Histamine, and PGD_2_

DNP-IgE-sensitized RBL-2H3 cells were pretreated with RVE for 1 h and then treated with DNP-HSA for 4 h. The concentrations of inflammatory mediators in culture medium were determined using enzyme-linked immunosorbent assay kits and microplate reader according to the manufacturer's instruction.

### 2.8. Western Blot Analysis

The cells were scraped from the plates with RIPA lysis buffer containing a protease and phosphatase-inhibitor cocktail (Roche, Basel, Switzerland). The lysates were quantified using the BCA protein assay kits (Thermo, MA, USA). Equal amounts of protein were resolved on 10% SDS-PAGE and then transferred to a PVDF membrane. Anti-COX-2, anti-*β*-actin, anti- phospho-Lyn, anti-phospho-Syk, anti-phospho-Fyn, anti-phospho-PLC*γ*1, anti-phospho-cPLA2, anti-phospho-ERK, and anti-phospho-Akt (Cell Signaling, MA, USA) were used to detect COX-2, *β*-actin and the phosphorylated form of Lyn, Syk, Fyn, PLC*γ*1, cPLA2, ERK, and Akt, respectively.

### 2.9. Animals

Male ICR mice, 5 weeks of age, were randomly assigned to five groups after 1 week adaptation period: control group (CTL, *n* = 5), Ag/IgE group (Ag/IgE, *n* = 5), Ag/IgE treated with 10 mg/kg dexamethasone group (Dex, *n* = 5), Ag/IgE treated with 250 mg/kg RVE group (RVE 250, *n* = 5), and Ag/IgE treated with 500 mg/kg RVE group (RVE 500, *n* = 5). RVE was prepared in saline, and CTL and Ag/IgE groups received equivalent volumes of saline. All experiments were approved by the Committee on Animal Experimentation and Ethics of KIOM.

### 2.10. Passive Cutaneous Anaphylaxis (PCA) in Mice

On day 1, anti-DNP-IgE (4 *μ*g/mL) antibody was subcutaneously injected into the ears of mice. On day 2, IgE-sensitized mice were administered with oral RVE (250 or 500 mg/kg) or dexamethasone (10 mg/kg). One hour later, DNP-HSA (300 *μ*g/mL) containing 1% Evans blue was injected into the tail veins. One hour later, the mice were anesthetized with CO_2_, and tissues from the treated ears were obtained. The Evans blue dye was removed by the ear tissue, which were then incubated with 0.4 mL formamide at 63°C for 16 h. Absorbance at 620 nm wavelength was measured using a microplate reader.

### 2.11. Statistical Analysis

The results are presented the mean ± SE of from three independent experiments. All data were statistically evaluated by one-way ANOVA with Bonferroni's post hoc test using Graph Pad PRISM software (Graph Pad PRISM software Inc., Version 5.02, LaJolla, CA, USA). Differences were considered statistically significant at ^*∗*^*p* < 0.05, ^*∗∗*^*p* < 0.005, and ^*∗∗∗*^*p* < 0.0005 between each treated group and the negative control (IgE/Ag group).

## 3. Results

### 3.1. HPLC Analysis of RVE

**Figure 1:**
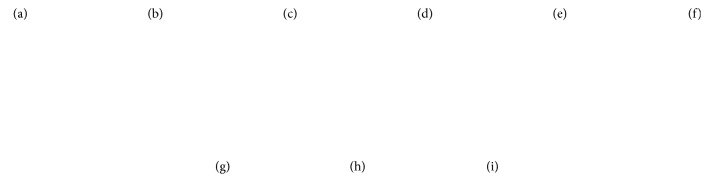
Chromatograms of seven components in *Rhus verniciflua* extract (RVE). Gallic acid (1), protocatechuic acid (2), methyl gallate (3), caffeic acid (4), ethyl gallate (5), quercetin (6), and butein (7) were identified at wavelengths of 254 nm (a) and 360 nm (b) using HPLC-DAD.

The identification of the seven components in RVE was based on comparisons of their retention times (tR), UV spectra, and chromatograms pattern with those of each standard using HPLC analysis system. As shown in Figure 1, the mixed seven standard components were well separated and showed good selectivity, without interference by other analytes within 70 min. The retention time of each standard component was analyzed at 4.81 min (1, gallic acid), 8.09 min (2, protocatechuic acid), 12.36 min (3, methyl gallate), 14.45 min (4, caffeic acid), 20.70 min (5, ethyl gallate), 55.58 min (6, quercetin), and 61.50 min (7, butein) in the chromatogram [13]. Under the same conditions, the retention times of the observed seven components in RVE were 4.81 min (1), 8.10 min (2), 12.36 min (3), 14.46 min (4), 20.71 min (5), 55.63 min (6), and 61.52 min (7), respectively. The UV wavelength of the seven components in RVE was optimized according to UV spectrum and the maximum absorption of each standard component. Analytes 1, 2, 3, and 5 were detected at 254 nm (Figure 1(a)), and 4, 6, and 7 were detected at 360 nm (Figure 1(b)).

### 3.2. RVE Inhibits IgE-Antigen-Mediated Degranulation in RBL-2H3 Mast Cells

**Figure 2:**
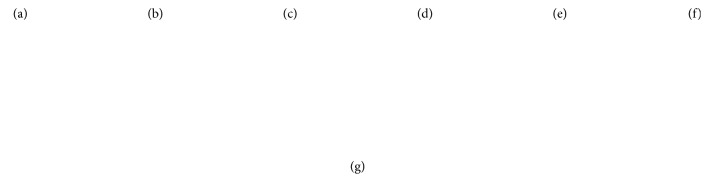
Effects of *Rhus verniciflua* extract (RVE) on (a) cell viability, (b) *β*-hexosaminidase, and inflammatory cytokines such as (c) TNF-*α*, (d) IL-4, and (e) IL-6 in RBL-2H3 mast cells. The results are expressed as the mean ± SE of at least three independent experimental results that were tested by the analysis of variance with Bonferroni's post hoc testing. ^#^*p* < 0.05 and ^###^*p* < 0.0005 versus the control group; ^*∗*^*p* < 0.05 and ^*∗∗∗*^*p* < 0.0005 versus the DNP-HSA-treated group. NS, not significant at the 0.05 probability level.

In order to determine any cell viability of RVE treatment on RBL-2H3 cells, we performed the MTT assay and found out that RVE did not show cytotoxicity at the concentration range of 100–500 *μ*g/ml in RBL-2H3 cells (Figure 2(a)). The *β*-hexosaminidase release use it as a marker of degranulation on IgE-antigen-mediated allergic reaction in mast cell [14]. RVE significantly decreased *β*-hexosaminidase release in a dose-dependent manner on IgE-antigen-stimulated RBL-2H3 cell (Figure 2(b)).

### 3.3. RVE Inhibits Proinflammatory Mediator Release in RBL-2H3 Mast Cell

IgE-antigen-induced mast cells secrete inflammatory cytokines, which are used as an indicator of immune and allergic responses [15]. To analyze inhibitory effect of RVE on inflammatory mediator release, we measured TNF-*α*, IL-4, and IL-6 in cell culture supernatant. The levels of TNF-*α* significantly showed a decreased tendency at 300 and 500 *μ*g/mL (Figure 2(c)). As shown in Figure 2(d) and 2(e), RVE significantly decreased the levels of IL-4 and IL-6 at all concentrations. In addition, we found that RVE inhibits secretion of cytokine better than dexamethasone.

### 3.4. RVE Inhibits the Fc*ε*RI Signaling Pathway in RBL-2H3 Mast Cells

**Figure 3:**
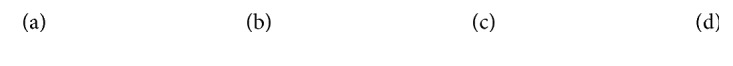
Effect of *Rhus verniciflua* extract (RVE) on the expression of Fc*ε*RI signaling pathway-related genes in RBL-2H3 mast cells. (a) Histamine levels in the culture medium of IgE-sensitized RBL-2H3 mast cells treated with RVE. (b, c) Immunoblot analysis performed with anti-p-Syk, -p-Lyn, -p-Fyn, -p-PLC*γ*1, -p-Akt, and -p-ERK antibodies. *α*-Tubulin was used as the protein loading control. Results are expressed as mean ± SE of at least five independent experimental results that were tested by analysis of variance with Bonferroni's post hoc testing; ^#^*p* < 0.005, ^##^*p* < 0.005, and ^###^*p* < 0.0005 versus the control group; ^*∗*^*p* < 0.05, ^*∗∗*^*p* < 0.005, and ^*∗∗∗*^*p* < 0.0005 versus the DNP-HSA-treated group.

We investigated concentration of histamine and expression of Fc*ε*RI signaling-related gene in early phase. Histamine is initial mediator of the allergic reaction in IgE-antigen-medicated mast cell [15], and histamine levels demonstrated a tendency to decrease at all concentrations (Figure 3(a); not significant). RVE pretreated cells were incubated with antigen for 10 min, and then we measured expression of Fc*ε*RI cascade-related proteins. The phosphorylation of Lyn was reduced at a 500 *μ*g/mL concentration of RVE. The phosphorylated form of Syk and Fyn decreased in a concentration-dependent manner in RVE-treated cells. Also, RVE remarkably reduced phosphorylation of PLC*γ*1. Furthermore, RVE significantly reduced phosphorylation of ERK, a mitogen-activated protein kinase, at 100 *μ*g/mL concentration, but no significantly effect at 300–500 *μ*g/mL concentration. The phosphorylated Akt was reduced by RVE in a concentration-dependent manner (Figures 3(b) and 3(c)). These results showed that RVE inhibits allergic response by inhibiting the Fc*ε*RI signaling pathway in early phase.

### 3.5. RVE Inhibits the Arachidonate Signaling Pathway in RBL-2H3 Mast Cells

**Figure 4:**
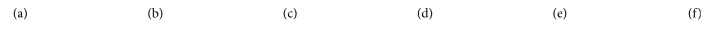
Effect of *Rhus verniciflua* extract (RVE) on the expression of COX-2 and p-cPLA2 genes in RBL-2H3 mast cells. (a) PGD_2_ levels in the culture medium of IgE-sensitized RBL-2H3 mast cells treated with RVE. (b, c) Immunoblot analysis performed with anti-COX-2 and p-cPLA2 antibodies. *α*-Tubulin was used as protein loading control. Results are expressed as mean ± SE of at least five independent experimental results that were tested by analysis of variance with Bonferroni's post hoc testing; ^#^*p* < 0.05 versus the control group; ^*∗*^*p* < 0.05, ^*∗*^*p* < 0.005, and ^*∗∗∗*^*p* < 0.0005 versus the DNP-HSA-treated group.

We analyzed the arachidonate signaling pathway in late phase by measuring levels of PDG_2_ and phosphorylation of COX-2 and cPLA2. PGD_2_ levels demonstrated a tendency to decrease at concentrations of 100 and 300 *μ*g/mL (Figure 4(a)). The expression of phosphorylated cPLA2 and COX-2 proteins was notably decreased in a dose-dependent manner (Figures 4(b) and 4(c)). It seems like that the allergic response reduced via preventing the late phase in IgE-antigen-activated mast cells.

### 3.6. Effect of RVE on Allergic Responses in the PCA Model

**Figure 5:**
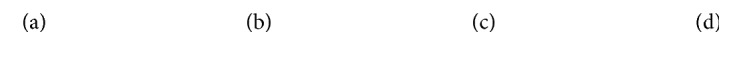
Effect of *Rhus verniciflua* extract (RVE) on the Ag/IgE-induced passive cutaneous anaphylaxis (PCA) model. ICR mice were subcutaneously injected with DNP-IgE (4 *μ*g/mL) into the ear. After 24 h, mice were orally administered with RVE (250 or 500 mg/mL) and dexamethasone (10 mg/mL). After 1 h, DNP–HSA (300 *μ*g/mL) containing 1% Evans blue was intravenously injected into their tail veins for 1 h The extravasated dye in the ears was analyzed using the procedure described in the Materials and Methods section. Results are expressed as mean ± SE of at least five independent experimental results that were tested by analysis of variance with Bonferroni's post hoc testing; ^###^*p* < 0.0005 versus the control group; ^*∗∗∗*^*p* < 0.0005 versus the DNP-Ag-treated group.

The concentration of Evans blue significantly increased from 3.69 ± 0.15 *μ*g/ear in the CTL group to 19.29 ± 2.47 *μ*g/ear in the Ag/IgE group with the PCA reaction (*p* < 0.0005). Concentrations of Evans blue were significantly lower in the RVE 250 group (6.35 ± 0.53 *μ*g/ear, *p* < 0.0005), the RVE 500 group (5.69 ± 0.90 *μ*g/ear, *p* < 0.0005), and Dex group (6.08 ± 0.85 *μ*g/ear, *p* < 0.0005) (Figure 5).

## 4. Discussion

The mast cell is a critical source of studies on inflammatory and allergic diseases such as allergic rhinitis, asthma, and autoimmune disease [16]. Mast cell secretes a number of inflammatory mediators like cytokines, histamine, PGD_2_, and leukotrienes from granules, and the mast cells have high affinity with Fc receptor for IgE on their cell surface [15]. RBL-2H3 cells, mast cell model, are mainly used for analyzing degranulation and allergic response [17]. In this study, we investigated that RVE effects on antiallergic reactions in IgE-antigen-mediated RBL-2H3 cells.

Antiallergic reactions of RVE result from its ability to suppress both the degranulation process and the production of inflammatory mediators (e.g., TNF-*α*, IL-4, IL-6, histamine, and PGD_2_) in IgE-antigen-mediated mast cells. *β*-Hexosaminidase is used as an indicator of degranulation and is useful for monitoring allergy inhibitory effects of unknown substances [18]. Consistent with the previous studies, we found that RVE inhibited degranulation in IgE-antigen-activated mast cells by depressing *β*-hexosaminidase releases. As undergoing degranulation, secretion of histamine and expression of Fc*ε*RI signaling-related protein promote release of the inflammatory cytokines such as TNF-*α*, IL-4, and IL-6 [19]. Cytokines are mainly secreted from T cells and are signaling substances that control and stimulate the immune system in the human body. In addition to the immune response, cytokines play an important role in the hematopoietic function, tissue regeneration, and development and growth of cells [15]. IL-4, involved in a late allergic response, modulates IgE-antigen-mediated immune and allergic responses due to enhancing adhesion molecule expression and cytokine production [20]. IL-4-deficient mice do not exhibit IgE response, but transgenic mice with increased IL-4 production exhibit high circulating IgE levels [21]. TNF-*α*, which is originally isolated in granules or is newly produced by activated mast cells themselves, is secreted by stimulating with IgE in mast cells. TNF-*α* released in mast cells affects the endothelial cells and mediates allergic inflammatory reaction. It is not only the inducer of another inflammatory cytokine but also a self-sustaining agent [22, 23]. IL-6 influences on the propagation of mast cells, excessive production of which is associated with chronic inflammatory diseases as well as with autoimmune disorders like rheumatoid arthritis and lupus nephritis [24]. The results of the present study suggest that RVE remarkably suppressed production of inflammatory cytokines on IgE-antigen-stimulated mast cell with respect to concentration.

To reveal the underlying mechanism of antiallergic response, we analyzed activation of both Fc*ε*RI signaling cascade-related kinase and MAP kinases, and they are important factors in early phase of allergic response [7]. The initial stage of signaling begins with the IgE receptor agglutination by the antigen, and then Lyn and Fyn of Src family kinases are phosphorylated by interacting with Fc*ε*RI [25, 26]. Next, the signaling pathway is initiated by activation of the ITAMs of the *β* and *γ* subunits of the Fc*ε*RI receptor by Lyn kinase. Syk is activated by attaching to the gamma subunit of the phosphorylated Fc*ε*RI receptor, activates the downstream signaling molecules such as Akt and PLC*γ*1, and elevates release of inflammatory mediators via activation of MAP kinases [27–29]. MAP kinases play a major role in multiple signaling of inflammation and apoptosis and regulate cytokine release including TNF-*α* and IL-4 due to extracellular stimulation in mast cells [30]. MAP kinase is a signaling system resulting in the activation of ERK, which involves transcription factors and stimulates gradual activation of enzymes of MAPKs [28]. We investigated the effect of RVE on ERK activation in Fc*ɛ*RI-mediated mast cells. The results showed no effect at 300 *μ*g/mL concentration, but it showed effect at 100 *μ*g/mL minimum and 500 *μ*g/mL maximum concentration. Based on these results, we thought that it would be effective enough for ERK activity. Phosphorylation of Akt and ERK plays an important role in a stimulus for mast cell degranulation in the early stages of an allergic reaction [28]. Activation of phospho-Akt and phospho-ERK by Fc*ε*RI-induced modulates production and secretion of TNF-*α*, and ERK sequentially activates enzymes of the MAPKs signaling system [30]. We suggest that RVE depressed activation of gene in early phase, thereby reducing the secretion of inflammatory cytokines and allergic reactions in IgE/Ag-stimulated mast cells.

Furthermore, RVE inhibited expression of COX-2 and cPLA2 and reduced the levels of PGD_2_, which is enhanced in activated immune cells, including mast cells. cPLA2 is a rate-limiting enzyme in the arachidonate cascade, producing arachidonic acid [31]. Arachidonic acid is metabolized into different lipid mediators, mainly through the COX-2 which is a rate-limiting enzyme for prostaglandin biosynthesis, producing prostaglandins such as PGD_2_, which are enhanced in activated immune cells, including mast cells [31, 32]. PGD_2_ is the major cyclooxygenase metabolite of arachidonate released by activated allergic response in mast cells [6]. The suppressive effects of RVE on PGD_2_ formation may contribute to its increased antiallergic activity, as PGD_2_ may mediate the allergic action associated with Fc*ɛ*RI-mediated allergic reaction [33]. This result suggests that RVE may inhibit IgE-antigen-mediated allergic reaction by downregulating COX-2 and cPLA_2_ and COX-2 product PGD_2_ levels.

Finally, we examined how RVE suppresses IgE-mediated PCA in mice. PCA is characterized by an immediate skin reaction at a localized IgE-mediated allergic response in vivo, typically with increased vascular leakage in the skin that can be assessed by an intravenous injection of Evans blue [34]. RVE successfully reduced allergic inflammatory responses in the PCA-induced mice.

## 5. Conclusions

In conclusion, we have shown that RVE reduces the risk of IgE-antigen-mediated allergic reactions by inhibiting degranulation in mast cells and modulating inflammatory cytokine release. RVE reduced Fc*ε*RI signaling-related gene expressions (e.g., Lyn, Syk, and Fyn) and extracellular signal-regulated kinase phosphorylation in mast cells (in early phase). Also, RVE reduced PGD_2_ release via regulation of COX-2 and cPLA_2_ in Fc*ɛ*RI-mediated mast cells (in late phase). RVE decreased the risk of allergic reactions related to the Fc*ɛ*RI-mediated process, which may be a potentially beneficial effect of antiallergic drugs.

## Data Availability

The datasets used and analyzed during the current study are available from corresponding author on reasonable request.
